# Characterization of highly expressed novel hub genes in hepatitis E virus chronicity in rabbits: a bioinformatics and experimental analysis

**DOI:** 10.1186/s12917-022-03337-x

**Published:** 2022-06-23

**Authors:** Manyu Li, Yan Wang, Kejian Li, Haiyun Lan, Cheng Zhou

**Affiliations:** 1 Division I of In Vitro Diagnostics for Infectious Diseases, Institute for In Vitro Diagnostics Control, National Institutes for Food and Drug Control, 2 Tiantanxili Rd, Dongcheng District, 100050 Beijing, China; 2 First Clinical Medical College, Shanxi Medical University, Taiyuan, China

**Keywords:** Hepatitis E virus, Chronic infection, Zoonosis

## Abstract

**Background:**

Hepatitis E virus (HEV), which is the leading cause of acute viral hepatitis worldwide, usually causes self-limited infections in common individuals. However, it can lead to chronic infection in immunocompromised individuals and its mechanisms remain unclear. Rabbits are the natural host of HEV, and chronic HEV infections have been observed in rabbits. Therefore, we aimed to investigate potential key genes in HEV chronicity process in rabbits. In this study, both bioinformatics and experimental analysis were performed to deepen the understanding of hub genes in HEV chronic infection in rabbits.

**Results:**

Ninety-four candidate differentially expressed genes (DEGs) and the pathways they enriched were identified to be related with HEV chronicity. A total of 10 hub genes were found by protein–protein interaction (PPI) network construction. Rabbits of group P (*n* = 4) which showed symptoms of chronic HEV infection were selected to be compared with HEV negative rabbits (group N, *n* = 6). By detecting the identified hub genes in groups P and N by real-time PCR, we found that the expressions of MX1, OAS2 and IFI44 were significantly higher in group P (*P* < 0.05).

**Conclusions:**

In this work, we presented that MX1, OAS2 and IFI44 were significantly upregulated in HEV chronic infected rabbits, indicating that they may be involved in the pathogenesis of HEV chronicity.

**Supplementary Information:**

The online version contains supplementary material available at 10.1186/s12917-022-03337-x.

## Background

Hepatitis E virus (HEV), which is the leading cause of acute viral hepatitis worldwide, can lead to 20 million HEV infections annually [1]. HEV is a positive-sense, single-stranded RNA virus with a 7.2 kb genome and belongs to the Hepeviridae family and the Orthohepevirus genus [2]. HEV has only one serotype, and can be mainly divided into 8 genotypes (HEV1-8) [1, 3], of which HEV1 and 2 only cause human infections and HEV3, 4, 7 and 8 have been recognized as zoonotic viruses [1, 4].

HEV usually causes self-limited HEV infections in common individuals [5]. However, HEV can establish chronic infections in immunocompromised individuals, including patients with solid organ transplant (SOT) [6], human immunodeficiency virus (HIV) infection [7] and hematological malignancies [8]. HEV chronic infections are mostly caused by HEV3 [9], and few cases have been observed with HEV4 [10] and 7 infection [11]. The specific mechanisms of HEV chronic infection are not well understood now and viral factors and host immunity may be related to HEV chronicity [5, 12]. It was demonstrated that a sustained stimulation of Th2 immunity was observed in chronic HEV infection animal models [13], and interferon-stimulated genes may be involved in the persistence of an HEV infection [14]. Still, further studies are warranted to clarify the molecular mechanisms underlying HEV chronic infection.

With the development of high-throughput technologies and bioinformatic analysis, the pathogenesis of many complex diseases can be investigated [15–17]. Hub genes are crucial genes that have interactions with other genes in gene networks and play an important role in the network, which are defined as the genes with connectivity greater than 10 in the interaction network [18, 19]. However, so far studies regarding gene expression profiling of chronicity of HEV are rare. As the natural host of HEV, rabbit models have been used to investigate the clinical symptoms and pathogenesis of HEV infection in several studies [20, 21]. Therefore, in this study, we aimed to identify differentially expressed genes (DEGs) and potential key genes in HEV chronicity process and further demonstrate these genes in animal models.

## Results

### Sample information processing and screening and identification of candidate differentially expressed genes (DEGs)

**Fig. 1:**
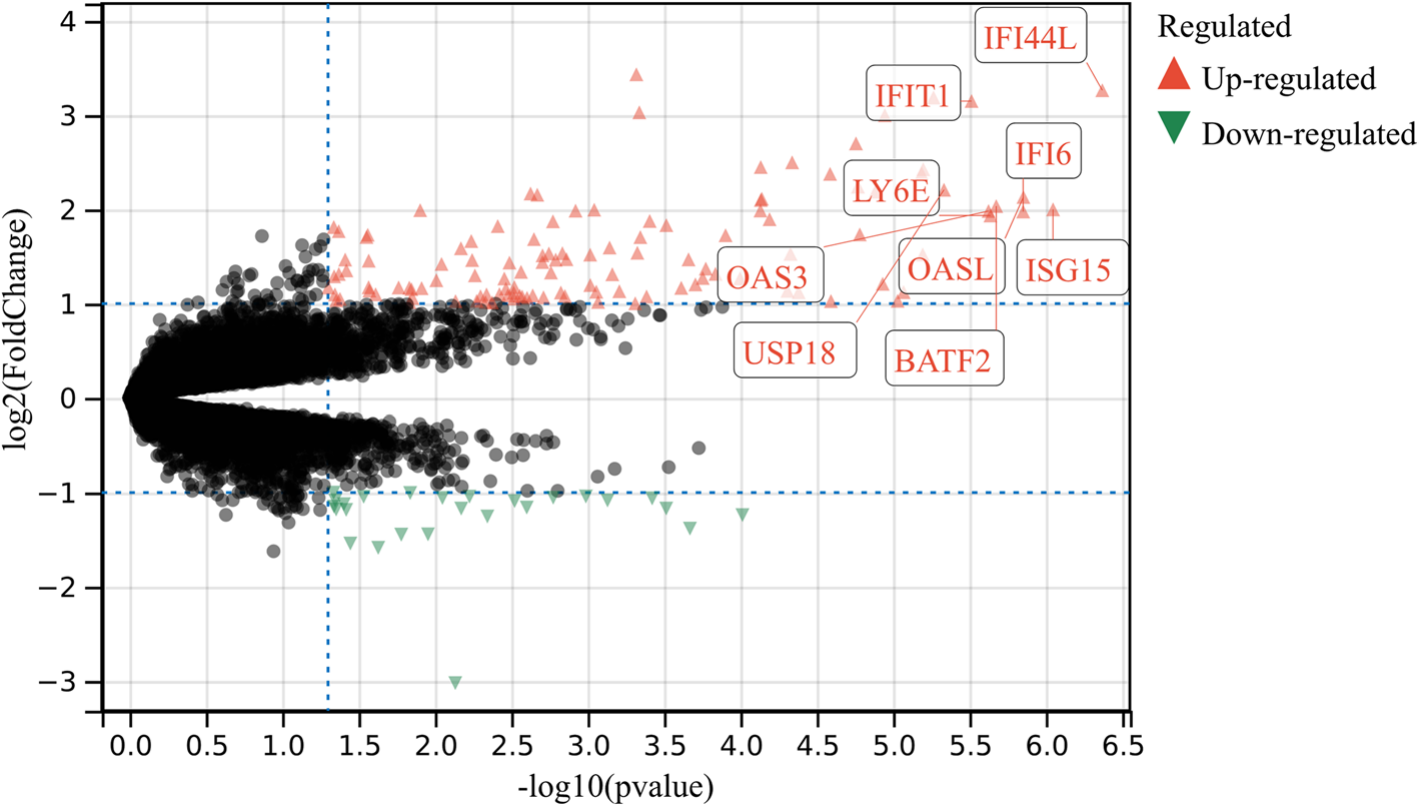
Volcano plotting of candidate DEGs between the whole blood of chronic HEV infection patients and control patients by bioinformatics analysis (*P* value < 0.05 and |log 2 FC|> 1)

The results of ‘R-limma’ analysis showed that there were 94 DEGs, including 80 upregulated DEGs and 14 downregulated DEGs in HEV chronic infected samples compared with normal samples (Fig. 1). The screening criteria for differentially expressed genes were as follows: adjust *P*-value < 0.05 and log twofold change > 1. Therefore, those DEGs were identified as candidate DEGs in this study.

### The enrichment analysis in HEV chronic infected samples

**Fig. 2:**
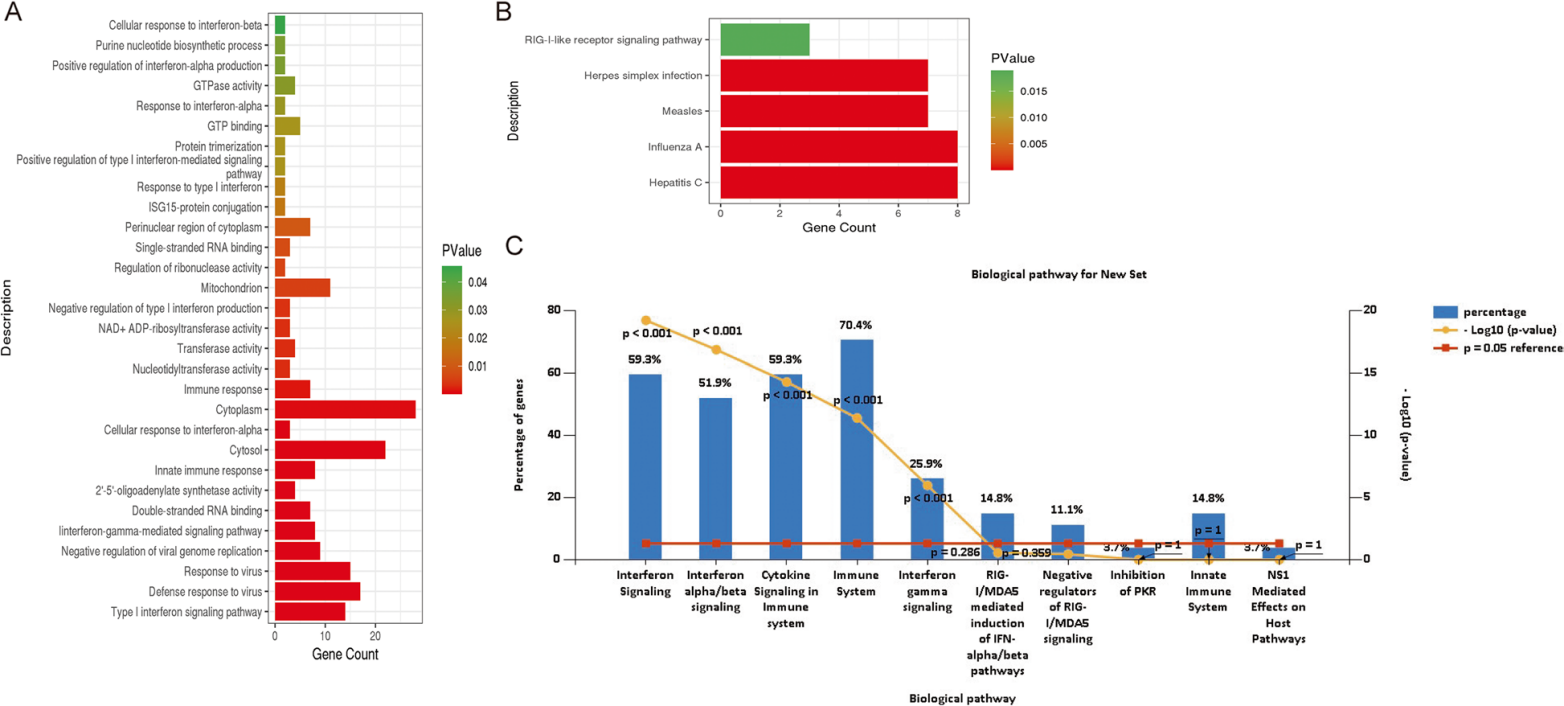
The enrichment analysis in HEV chronic infected samples. **A** GO terms of targets of DEGs. **B** Significantly infuenced KEGG pathways by enrichment analysis. **C** Enrichment analysis by Funrich

In the enrichment analysis, DAVID and Fun Rich software were used of the samples’ genes. The GO terms of targets of DEGs were shown in Fig. 2A. The DEGs enriched in the BP terms were mostly associated with type I interferon signaling pathway, defense response to virus, negative regulation of viral genome replication, response to virus and interferon-gamma-mediated signaling pathway; those in the CC category mostly included the cytosol, cytoplasm and mitochondrion; and those belonged to MF category were mainly related to 2’-5’-oligoadenylate synthetase activity, nucleotidyltransferase activity and transferase activity. The KEGG pathway enrichment analysis revealed that the targets of DEGs were enriched in pathways in hepatitis C, influenza A, measles 7, herpes simplex infection and RIG-I-like receptor signaling pathway. The top 5 results of the KEGG pathway enrichment analysis are shown in Fig. 2B.

Then the DEGs were uploaded to Funrich for further enrichment analysis. By the analysis, the results indicated that DEGs in HEV chronic infected samples were mainly enriched in interferon signaling pathway and immune-related pathways (Fig. 2C).

### Protein–protein interaction (PPI) network construction and hub genes identification

**Fig. 3:**
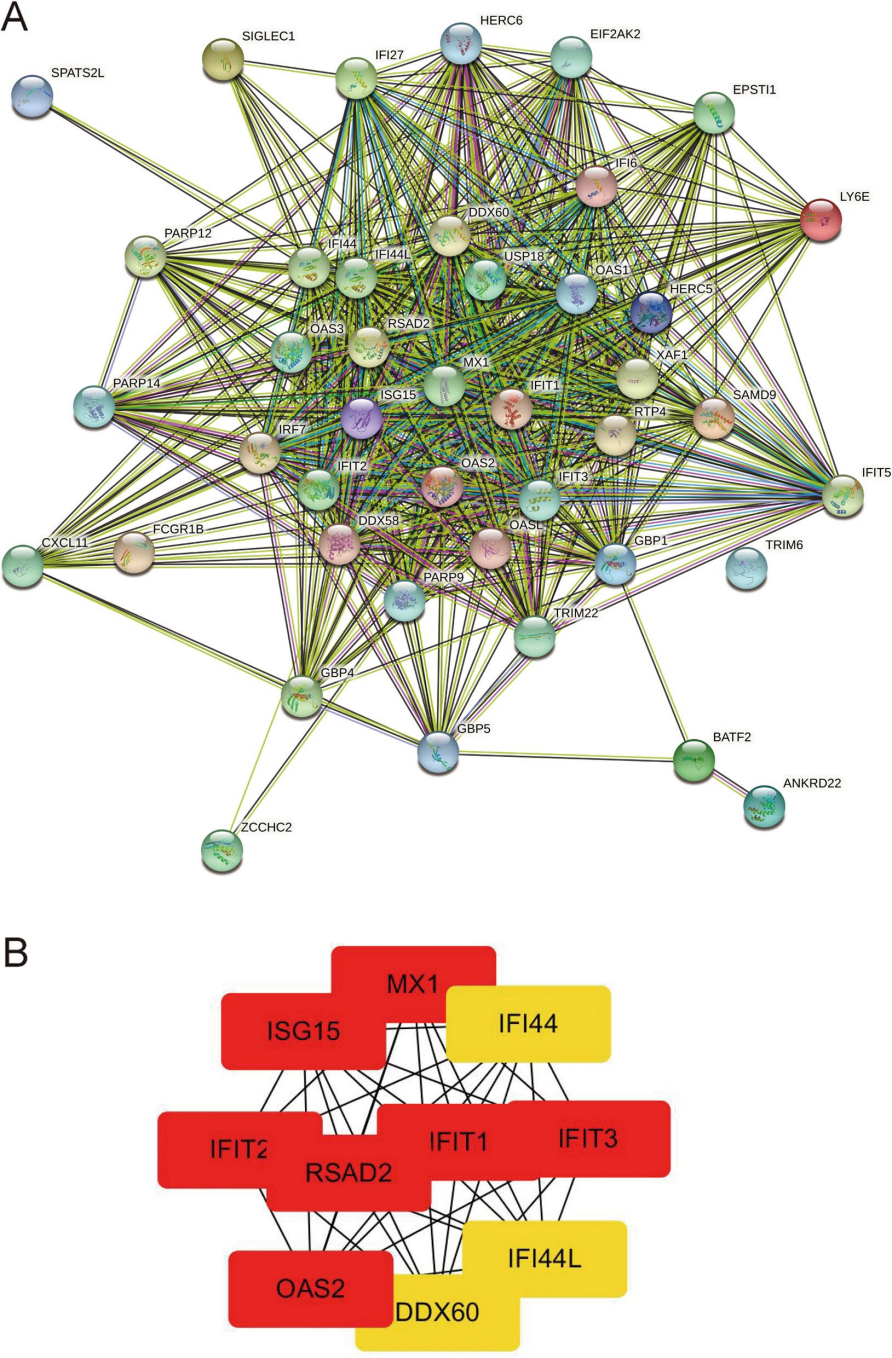
The PPI network construction and hub genes identification. **A** PPI network was constructed on the basis of the STRING database. **B** 10 hub genes of HEV chronic infected samples were identified

PPI network which contained 549 nodes was then constructed by Cytoscape software on the basis of the STRING database to further screen the hub genes among the DEGs (Fig. 3A). MCODE was used to carry out the network data analysis to identify gene clusters, genes in the first gene cluster with the highest score were selected for BP enrichment analysis, and the results showed that these genes in the first gene cluster were mostly involved in defense response to virus and immune response functions. Then, 10 hub genes of HEV chronic infected samples were selected (Fig. 3B).

### Pathogenicity of HEV chronic infection in rabbits

**Fig. 4:**
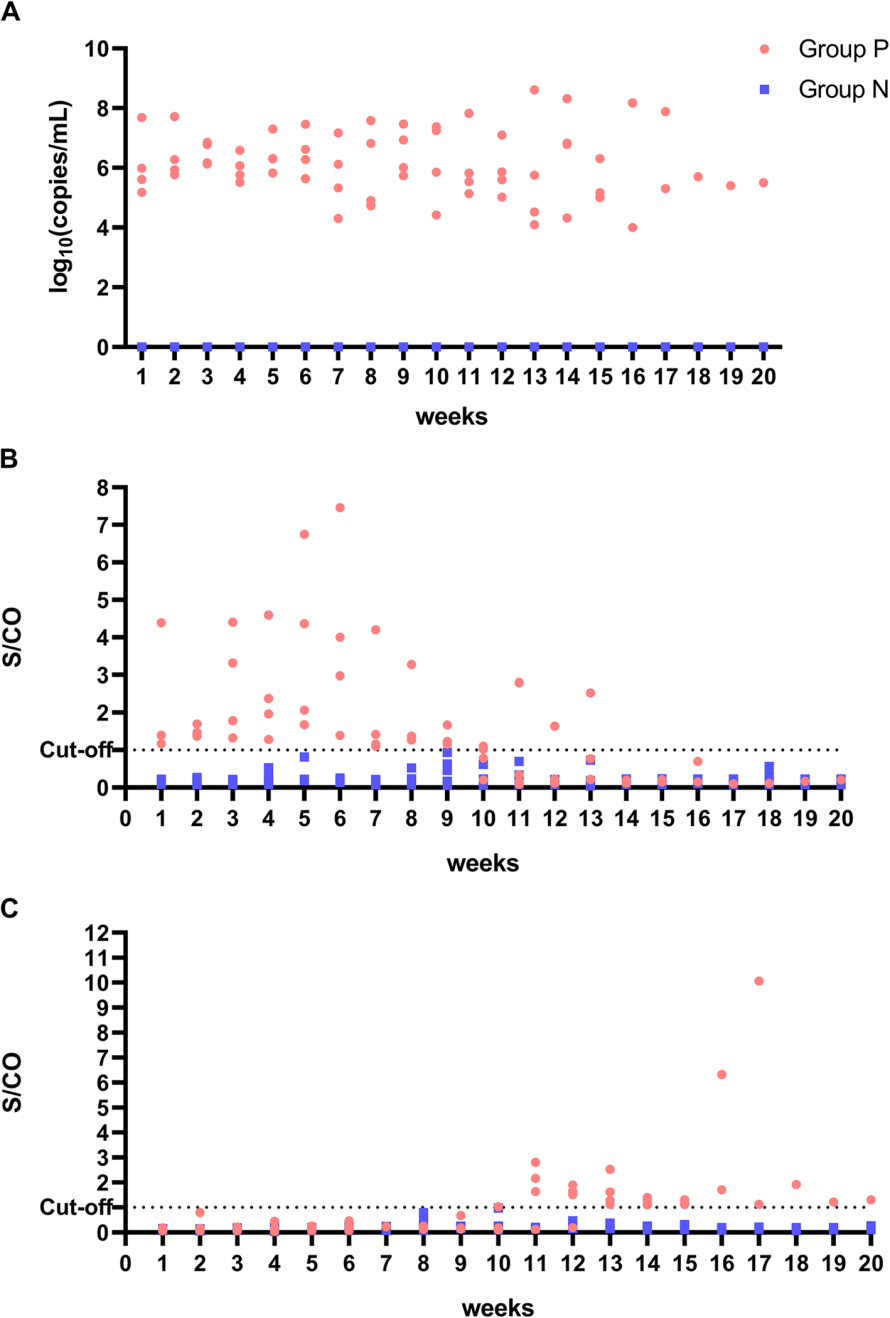
The characteristics of HEV chronic infection in rabbits. **A** The fecal viral load levels of groups P and N. **B** Serum HEV antigen dynamic changes of groups P and N. **C** Anti-HEV antibody levels of groups P and N

The duration of fecal virus shedding of P1, P2, P3 and P4 were 14th, 17th, 15th and 20th weeks, respectively. The fecal viral load of group P was 10^4^–10^8^ (Fig. 4A). Serum HEV antigen of P1, P2, P3 and P4 was continuously positive before 9th, 10th, 9th and 13th weeks, respectively (Fig. 4B). The anti-HEV antibody of P1, P2, P3 and P4 showed seroconversion for anti-HEV antibody at 11th, 13th, 11th and 10th week, respectively (Fig. 4C). Group N, the negative control, showed no symptoms of HEV infection during the study.

### Identification of hub genes in chronic HEV infected rabbits

**Fig. 5:**
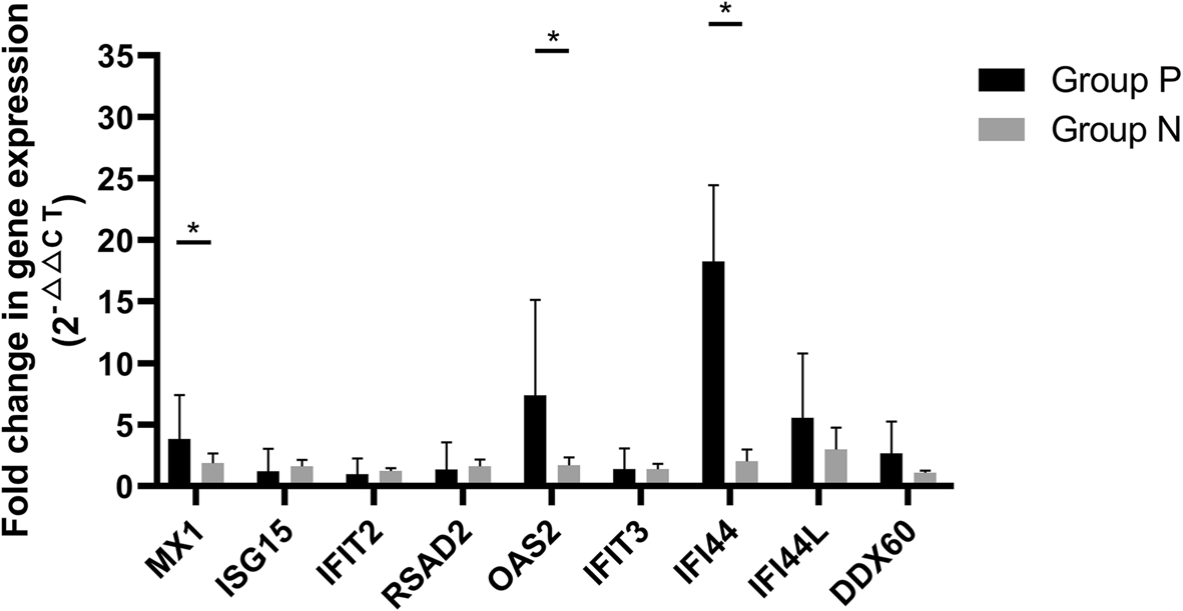
The comparison of hub genes expressions in rabbits. The expressions of MX1, ISG15, IFIT2, RSAD2, OAS2, IFIT3, IFI44, IFI44L and DDX60 were measured by real-time PCR. (*, *P* value < 0.05)

The rabbits of group P were euthanized 2 weeks after their virus fecal shedding ceased and the rabbits of group N were euthanized at the end of study. Their livers were collected and the RNA was extracted as described above. The hub genes identified in this study were quantified and compared between two groups (Fig. 5). The results showed that MX1, OAS2 and IFI44 were significantly up-regulated in HEV chronic infected rabbits (*P* < 0.05). Also, the expressions of these genes in positive dropouts (*n* = 7) were assessed (Supplementary Fig. 1), and the expressions of MX1, ISG15, RSAD2, OAS2, IFI44, IFI44L and DDX60 had significant differences in positive dropouts (*P* < 0.05).

## Discussion

So far, the validation of large independent datasets for HEV chronicity still remains rare. In this study, we aimed to identify potential key genes by the way of a series of bioinformatics analyses and experimental validation.

The bioinformatics analysis results showed that inflammatory- or immune response-related genes and pathways were involved in HEV chronicity. The enrichment analysis showed that DEGs were significantly enriched in the categories of cellular responses to interferon-β. KEGG pathway analysis showed that DEGs were significantly enriched in RIG-I-like receptor signaling pathway. Both innate and adaptive immune responses seem to play a robust role in HEV chronicity ([12]. Studies have shown that chronic hepatitis E (CHE) patients had lower levels of IL-1 receptor agonist and TNF-α concentration [12], and their total counts of CD2, CD3 and CD4 T cells were also significantly lower [22].

Specifically, 10 potential hub genes were selected in this study, including MX1, ISG15, RSAD2, OAS2, IFIT1, IFIT2, IFIT3, IFI44, IFI44L and DDX60. Some of these interferon-stimulated genes (ISGs) have been shown to be related with HEV infection. To verify the expressions of these genes in HEV chronically infected rabbits, we performed the animal experiments. Chronic HEV infection was observed in rabbits of group P, which was manifested by continuous fecal virus shedding for more than 12 weeks, the HEV antigen positivity and seroconversion for anti-HEV antibody. By real-time RT-PCR, we found that the levels of MX1, OAS2 and IFI44 were significantly higher in group P. However, the expressions of remaining genes didn’t show significant difference. Therefore, these results indicated that MX1, OAS2 and IFI44may play key roles in the pathogenesis of HEV chronic infection of rabbits. MX1 has been reported to inhibit the multiplication of many RNA and DNA viruses and were found to be upregulated in HEV infected patients [23, 24]. OAS2 is an interferon-stimulated antiviral enzyme and may be involved in HEV infection [25]. FI44 is an interferon-alfa inducible protein and is related with infection of several viruses, including hepatitis C virus (HCV), human papillomavirus (HPV) and human immunodeficiency virus (HIV) [26–28]. Our results showed that these gene was upregulated during the HEV chronic infection, indicating their potential role in HEV chronicity of rabbits.

## Conclusion

In conclusion, a total of 94 DEGs and 10 hub genes were identified by bioinformatics analysis. These DEGs in HEV chronic infected samples were mainly enriched in interferon signaling pathway and immune-related pathways. By experimental reserach, the present study demonstrated that hub genes, including MX1, OAS2 and IFI44, were involved in the pathogenesis of chronic HEV infection, indicating that these genes have the potential to be biomarkers in HEV chronicity. Further studies to confirm the mechanisms of these key genes and pathways are needed.

## Methods

### Acquisition of microarray data

The microarray data obtained from the gene expression profiling for chronic HEV infection were downloaded from the Gene Expression Omnibus database (https://www.ncbi.nlm.nih.gov/gds/). The dataset GSE36539, based on the GPL6480 platform, consists of the results from 10 whole blood of patients with chronic hepatitis E infection and 6 whole blood of control patients.

### Screening of candidate DEGs

To screen differentially expressed DEGs between the whole blood of chronic HEV infection patients and control patients, a method named “R-limma” package of Bioconductor (http://www.bioconductor.org/) and software R (version 3.4.4, https://www.r-project.org/) was introduced. Probe sets with a *P* value < 0.05 and |log 2 FC|> 1 were between two groups were identified as significant DEG sets.

### Functional annotation and pathway enrichment analysis

In this study, DAVID website (http://david.ncifcrf.gov/home.jsp) and Funrich software (http://www.funrich.org/) were used to evaluate the enrichment of the DEGs into the Cell Components (CC), Molecular Functions (MF), and Biological Processes (BP) of Gene Ontology (GO) terms. The Kyoto Encyclopedia of Genes and Genomes (KEGG) pathway enrichment analysis was performed to analyze relevant signaling pathways of DEGs [29]. *P*-value < 0.05 was regarded as statistically significant.

### Protein–protein interaction (PPI) network construction

The PPI network of DEGs was constructed by STRING database (http://string-db.org). Then, the hub genes in this network were identified by analyzing the degree of connectivity through Cytoscape software (version 3.6.1).

### Experimental design of animal study

Fifty 3-month-old Japanese white rabbits (weighing about 2–3 kg) obtained from the Department of Laboratory Animal Science of National Institutes for Food and Drug Control were randomly selected. Fecal and serum specimens of these rabbits were collected weekly for two consecutive weeks. All rabbits with positive fecal HEV RNA (*n* = 11) were selected for follow-up monitoring. Serum and fecal samples of the rabbits were collected weekly during the follow-up monitoring for HEV RNA, anti-HEV antibody, HEV antigen and alanine transaminase (ALT) level detection. Of all the 11 positive rabbits, 4 had fecal virus shedding for more than 12 weeks, which can be recognized as HEV chronic infection and classified into group P. Also, six 3-month-old Japanese white rabbits (weighing about 2–3 kg) with negative for fecal and serum HEV RNA and anti-HEV antibody were selected and classified into group N as a negative control. The animal experiments were approved by the Committee of Laboratory Animal Welfare and Ethics of National Institutes for Food and Drug Control (2022B012) and First Clinical Medical College, Shanxi Medical University (SYXK2019-0007).

### Sample collection

Feces were homogenized in sterile phosphate-buffered saline (PBS) to make 10% fecal suspensions (wt/vol). The suspensions were then clarified by centrifugation. The dissection and collection of tissue samples was conducted following standard protocols. Serum, fecal suspension and tissue samples were stored at − 80 °C for detection.

### Detection of anti-HEV, HEV antigen and HEV RNA

All rabbit serum samples were detected by ELISA for total anti-HEV antibody and HEV antigen according to the manufacturer’s instructions (Wantai, Biopharmaceutical, Beijing, China). Total RNA in fecal suspension and tissues was extracted by using QIAamp Viral RNA Mini Kit (Qiagen, Hilden, Germany), according to the protocol of the manufacturer. Real-time fluorescence quantitative PCR (RT-qPCR) in this study was performed by using the Taqman probe detection method and QuantiTect® Probe RT–PCR Kit (Qiagen, Hilden, Germany) [30].

### Detection of hub genes in rabbits

The sequences of rabbit MX1, ISG15, IFIT2, RSAD2, OAS2, IFIT3, IFI44, IFI44L and DDX60 were obtained from GenBank (Accession numbers XM_008252473, XM_017340429, XM_002718372, XM_017350632, XM_002719777, XM_008270166, XM_017346152, XM_008265011, XM_008249585, respectively), as was the sequence for glyceraldehyde-3-phosphate dehydrogenase (GAPDH) (Accession number NM_001082253) (used as a housekeeping gene for normalization). IFIT1 was not detected due to the lack of GenBank sequence. Using these sequences, primers were designed for each of the cytokines (Table 1) for hub gene quantitation by real-time RT-PCR. Real-time PCR assay was performed on an ABI Prism 7500 Sequence Detection System (Applied Biosystems, Foster City, CA, USA) using QuantiTect SYBR Green PCR Kit (Qiagen, Hilden, Germany).

**Table 1:** Primer information for hub genes detection

Gene	Forward primer	Reverse primer
MX1	5’- ATCAAGCAGAACCACGAGCA-3’	5’- CTTCTCGATCACGCTCTCCC-3’
ISG15	5’- GAAGTGTGACAACCCGCTGA-3’	5’- AAGCTCAGCCAGAACAGGTC -3’
IFIT2	5’- AGAAGGAGAAGGCGAGAGGT -3’	5’- GGAGGTAGGTGTTGTCTGGC -3’
RSAD2	5’- AAGAGGAGGAGGACAGCGAT -3’	5’- CAGGAGATGGCGAGGATGTC -3’
OAS2	5’- GAAGGAGCCCCAGGAGTTTC -3’	5’- CGAGGATCCTGAGCATGTCC -3’
IFIT3	5’- GAACAGAAACCGCACAACCC -3’	5’- AACTGCTCACCCTCAGCTTC -3’
IFI44	5’- AGCAGCTCCAGACCTTGTTC -3’	5’- AATCCGTGGACACTTGCCTT -3’
IFI44L	5’- TTTCAGAGGCCGTCCAACTC -3’	5’- TGCAGTCCCATTGTGTCACA -3’
DDX60	5’- GTGCGCGTACTTCAACTTCC -3’	5’- GGTTCAGGCCTTCATCTGCA -3’
GAPDH	5’- CGATGCCCCCATGTTTGTGA -3’	5’- TCATGAGCCCCTCCACAATG -3’

### Statistical analysis

Statistical analysis was performed using the SPSS PASW Statistics v18.0 statistical software package (SPSS, Inc., USA, http://www.ibm.com/cn/). Data were compared using Student’s t-test or chi-squared test. A *P*-value of < 0.05 was considered significant.

## Supplementary Information

**Additional file 1:** **Supplementary Figure 1.**The comparison of hub genes expressions in positive dropouts and negative rabbits. The expressions of MX1, ISG15, IFIT2, RSAD2, OAS2, IFIT3, IFI44, IFI44L and DDX60 were measured by real-time PCR. (*, *P*value < 0.05).

## Data Availability

The data shown in this paper are available within the article. The raw data of RNA- sequencing are available from the NCBI database under accession number GSE36539.

## Abbreviations

HEV: Hepatitis E virus
SOT: Solid organ transplant
HIV: Human immunodeficiency virus
DEG: Differentially expressed gene
CC: Cell components
MF: Molecular functions
BP: Biological processes
GO: Gene ontology (GO)
KEGG: Kyoto Encyclopedia of Genes and Genomes
PBS: Phosphate-buffered saline
CHE: Chronic hepatitis E
HCV: Hepatitis C virus
HPV: Human papillomavirus
ALT: Alanine transaminase
