# Supplementary figures and images for: Characterization of highly expressed novel hub genes in hepatitis E virus chronicity in rabbits: a bioinformatics and experimental analysis
Source: BMC Vet Res. 2022 Jun 23;18:239. doi: 10.1186/s12917-022-03337-x (PMC9219159; doi:10.1186/s12917-022-03337-x)

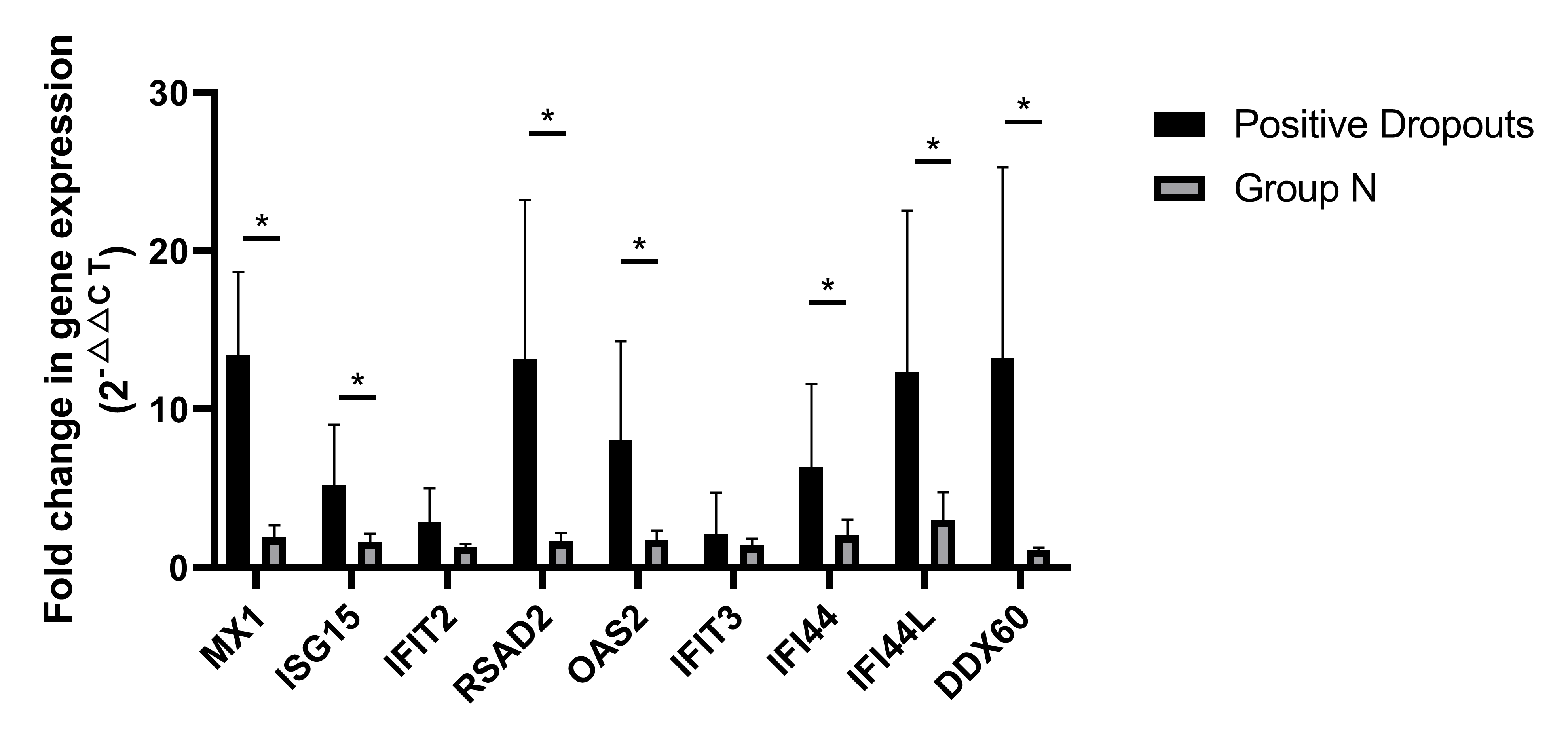

Supplement: Supplementary file 1 — Additional file 1: Supplementary Figure 1.The comparison of hub genes expressions in positive dropouts and negative rabbits. The expressions of MX1, ISG15, IFIT2, RSAD2, OAS2, IFIT3, IFI44, IFI44L and DDX60 were measured by real-time PCR. (*, Pvalue < 0.05). [file 12917_2022_3337_MOESM1_ESM.tif]
